# Iodine Images in Dual-energy CT: Detection of Hepatic Steatosis by Quantitative Iodine Concentration Values

**DOI:** 10.1007/s10278-022-00682-z

**Published:** 2022-07-25

**Authors:** Stefanie Beck, Laurenz Jahn, Dominik Deniffel, Isabelle Riederer, Andreas Sauter, Marcus R. Makowski, Daniela Pfeiffer

**Affiliations:** 1 Department of Diagnostic and Interventional Radiology, Klinikum Rechts der Isar, Technische Universität München, Ismaninger Str. 22, Munich, 81675 Germany; 2 Department of Diagnostic and Interventional Neuroradiology, Klinikum Rechts der Isar, Technische Universität München, Ismaninger Str. 22, Munich, 81675 Germany; 3 Department of Diagnostic and Interventional Neuroradiology, University Hospital RWTH Aachen, Pauwelsstr. 30, Aachen, 52074 Germany; 4 Institute for Advanced Study, Technical University of Munich, Lichtenbergstr. 2 a, Garching, 85748 Germany

**Keywords:** X-ray computed tomography, Dual-energy CT, Quantitative imaging, Iodine, Contrast media, Fatty liver

## Abstract

Hepatic steatosis is a common condition and an early manifestation of a systemic metabolic syndrome. As of today, there is no broadly accepted method for the diagnosis of hepatic steatosis in contrast-enhanced CT images. This retrospective study evaluates the potential of quantitative iodine values in portal venous phase iodine images in dual-energy CT (DECT) by measuring iodine concentrations in regions of interest (ROI) and analyzing the absolute iodine concentration of the liver parenchyma as well as three different blood-normalized iodine concentrations in a study cohort of 251 patients. An independent two sample *t*-test (*p* < 0.05) was used to compare the iodine concentrations of healthy and fatty liver. Diagnostic performance was assessed by ROC (receiver operating characteristic) curve analysis. The results showed significant differences between the average iodine concentration of healthy and fatty liver parenchyma for the absolute and for the blood-normalized iodine concentrations. The study concludes that the iodine uptake of the liver parenchyma is impaired by hepatic steatosis, and that the measurement of iodine concentration can provide a suitable method for the detection of hepatic steatosis in quantitative iodine images. Suitable thresholds of quantitative iodine concentration values for the diagnosis of hepatic steatosis are provided.

## Introduction

Hepatic steatosis is a common condition affecting 10–50% of the general population [1–4]. Nonalcoholic hepatosteatosis is considered the hepatic manifestation of a systemic metabolic syndrome including obesity, hyperlipidemia, type 2 diabetes, and hypertension and a potential cause of hepatic cirrhosis [5–10]. Timely diagnosis of hepatic steatosis and referral for medical intervention or changes in lifestyle are of critical importance.

The diagnosis of hepatic steatosis is often made incidentally on imaging studies performed for other purposes. Unenhanced CT imaging is a widely accepted standard to identify hepatic steatosis. Contrast-enhanced CT images are more controversially discussed for this purpose, and this method is often considered to be less precise [11–15], although some studies suggest the contrary [16, 17]. Kim et al. presented a study that compared unenhanced and contrast-enhanced CT images for the diagnosis of fatty liver disease using same-day liver biopsy as a reference standard [16] and concluded that contrast-enhanced CT images had an equal or even higher accuracy than unenhanced CT images for this purpose.

The technology of dual-energy computed tomography (DECT) offers a significant advantage over traditional single-energy CT systems [18–22]. By using two independent energy sets to examine differing attenuation properties, it allows to detect and quantify the amount of iodine contrast agent with high accuracy, showing maximum deviations of 5–10% between the measured and the actual iodine concentration in the analyzed tissues in previous studies [23–26].

Quantitative measurement of iodine concentration in different organs during clinical routine CT imaging offers a wide range of potential diagnostic applications. Previous studies have reported the diagnostic value of DECT-derived iodine concentration for the evaluation of pulmonary diseases [27], renal masses [28], lymphadenopathy [29], thyroid nodules [30], and myocardial perfusion [31].

In this study, we hypothesized that the direct quantification of iodine uptake may also offer additional diagnostic benefit to CT examinations of the liver tissue, providing a new approach to the detection of hepatic steatosis in contrast enhanced CT images. The purpose of this study was to evaluate the diagnostic potential of iodine concentrations for the diagnosis of hepatic steatosis and to define quantitative thresholds for the identification of fatty liver disease.

## Methods

### Study Population

The study was approved by the local ethics committee under IRB number 127/17S, and it was conducted in accordance with the guidelines of this board. Informed consent was waived given a retrospective study and anonymized patient data.

**Fig. 1:**
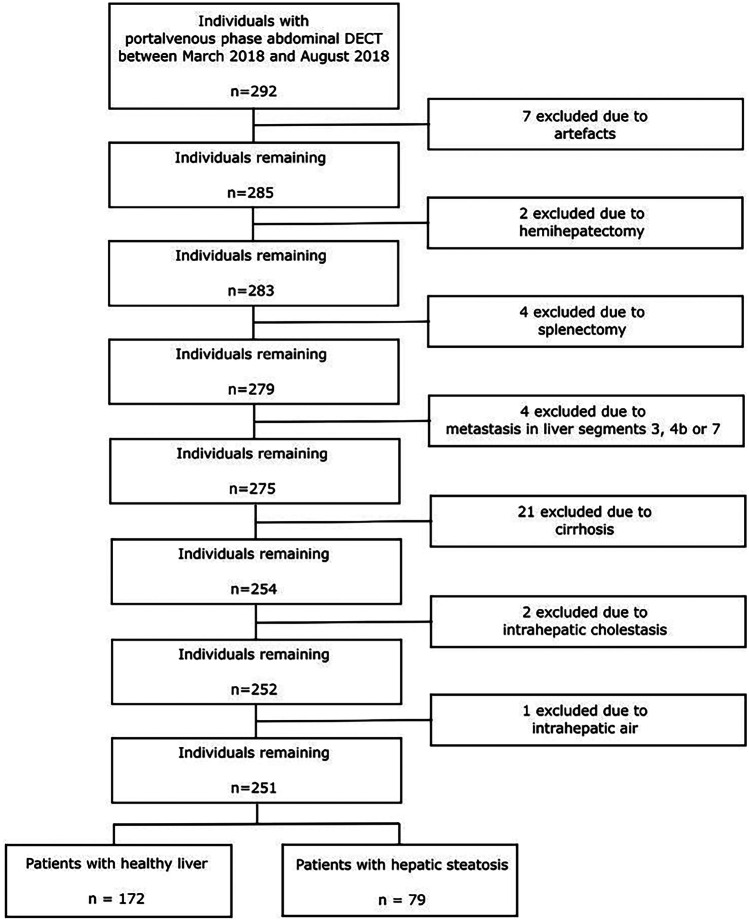
Flow diagram of patient inclusion

The computerized clinical database was retrospectively queried for all contrast-enhanced, portal venous phase abdominal DECT with quantitative iodine images generated between March 2019 and August 2019 in consecutive series. The inclusion was limited to a single CT examination per patient. Inclusion criteria were (a) contrast-enhanced portal venous phase DECT of the abdomen and (b) age > 18 years. The exclusion criteria were (a) poor image quality due to artifacts and (b) alternative diagnoses potentially affecting the iodine concentration measurements (hemihepatectomy, splenectomy, metastasis in measured liver segments, cirrhosis, intrahepatic cholestasis, and intrahepatic air) (Fig. 1).

The images of the final study population were categorized into healthy liver and hepatic steatosis. The reference standard used for the diagnosis of hepatic steatosis was an attenuation difference between the liver and the spleen tissue of at least 19 HU in contrast-enhanced portal venous phase images, following the suggested optimal cutoffs proposed by Kim et al. [16]. The diagnosis of healthy liver and hepatic steatosis were made independently by 2 radiologists with 7 and 12 years of experience interpreting abdominal CT. Pre-existing diagnostic findings were blinded.

### CT Protocol

A dual-layer dual-energy 64-channel CT scanner (IQon, Philips Healthcare, Cleveland, OH, USA) was used for the acquisition of all CT examinations of the abdomen according to our institutional protocol. A 20-G catheter and a dual-syringe injection system (Stellant, MEDRAD, Indianola, Pennsylvania) were used to apply a contrast agent (bolus of 80 ml Ultravist 370 MCT, Bayer Vital GmbH, Leverkusen, Germany). The contrast agent was administered into an ante-cubital vein with a rate of 3 ml/s, followed by a 50-ml saline chaser, and the portal venous phase images were obtained 70 s after the injection. All patients were scanned craniocaudally with a pitch of 0.9, a tube voltage of 120 kVp, and a 64 × 0.625 mm detector configuration. The reconstruction of the data sets was made in axial view using a 512-image matrix and slice thickness of 5 mm.

### CT Image Analysis

A commercially available spectral workstation (IntelliSpace Portal (v.8.0.2), Philips Healthcare, USA) was used for the image analysis. Previous studies have shown the high accuracy of the measurements with this workstation type [32].

The CT attenuation of the liver and the spleen tissue used as a reference for the diagnosis of hepatic steatosis was measured in 5-mm-thick portal-venous phase contrast-enhanced images by placing a total of 3 regions of interest (ROIs) of 1.5 cm^2^ each in the liver and one ROI of 1.5 cm^2^ in the spleen. Two of the liver ROIs were placed in the right hepatic lobe in segments 4b and 7, and one ROI was placed in the left hepatic lobe in segment 3. The average attenuation of the three ROIs placed in the liver was used as liver attenuation. Macroscopic hepatic vessels were carefully avoided during the placement of all ROIs.

**Fig. 2:**
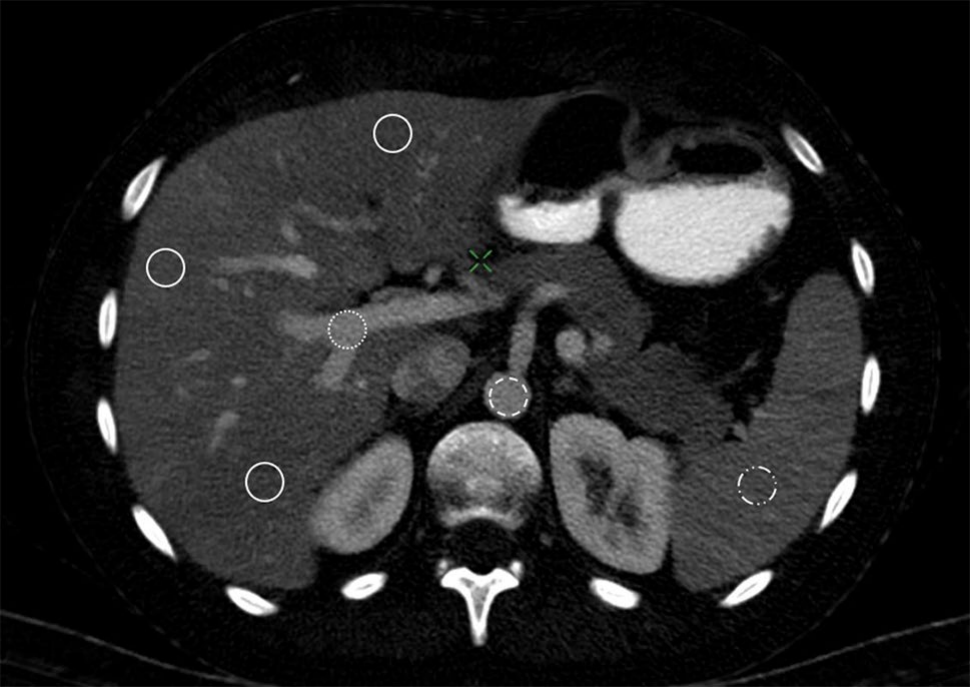
Placement of the ROIs in a transversal plane dual-energy CT quantitative iodine image of a 55-year-old male individual. The portal venous phase DECT iodine image corresponds to the coeliac axis and shows a total of six ROIs. Three ROIs are placed in the hepatic tissue (continuous line), one in segment 3 in the left hepatic lobe and two in the right hepatic lobe in segments 4b and 7. One ROI (dotted line) is in the portal vein, and one ROI (dashed line) is in the aorta. Another ROI is placed in the spleen tissue (dotted and dashed line)

The iodine concentration of the liver parenchyma was measured using the same three ROIs in 5-mm-thick portal-venous iodine images, assuring an exact match of the measured areas. Two further ROIs for the measurement of the iodine concentration were placed in the aorta, and portal vein, both at the level of the coeliac axis. The diameter of these two ROIs was adapted to the vessel lumina, enclosing the largest possible area, respectively. The positioning of all ROIs is shown in Fig. 2. All measurements were performed by an experienced radiologist, who was blinded to pre-existing clinical information.

The iodine concentration measured in the ROIs of the liver parenchyma will be referred to as *I*_*III*_, *I*_*IVb*_, and *I*_*VII*_, respectively, and the iodine concentrations measured in the aorta and portal vein will be referred to as *I*_*aorta*_ and *I*_*portalvein*_.

### Definition of Absolute Iodine Concentration

We defined the absolute iodine concentration of a patient *I*_*abs*_ as the average measured iodine concentration of the three liver parenchyma ROIs.

1 $${I}_{abs}\left(x\right)=\frac{{I}_{III }\left(x\right)+{I}_{IVb}(x)+{I}_{VII}(x)}{3}$$

The absolute iodine concentration was compared between healthy liver parenchyma and liver parenchyma affected by hepatic steatosis.

### Calculation of Blood-normalized Iodine Concentrations

Since we expected the iodine concentration of the blood-supplying vessels to have a major impact on the measured absolute iodine concentration of the liver parenchyma, we defined blood-normalized iodine concentrations *I*_*bn*_, by normalizing the iodine concentration measured in the liver parenchyma *I*_*abs*_ to the iodine concentration measured in the blood supplying vessels *I*_*blood*_.

For this matter, the absolute iodine concentration of the liver of each patient *I*_*abs*_(*x*) was weighted with the measured iodine concentration of the supplying vessels of the patient *I*_*blood*_(*x*). The resulting value was multiplied with the average blood iodine concentration over all patients’ *ØI*_*blood*_ to avoid losing the measuring units (mg/ml) through the equation.

2 $${I}_{bn}\left(x\right)=\frac{{I}_{\mathrm{abs}}(x)}{{I}_{\mathrm{blood}}(x)}*\varnothing {I}_{\mathrm{blood}}=\frac{{ I}_{\mathrm{abs}}\left(x\right)}{{I}_{\mathrm{blood}}\left(x\right)}* \frac{{\sum }_{i=1}^{n}{I}_{\mathrm{blood}}\left(i\right)}{n}$$

We evaluated three different blood-normalized iodine concentrations, with three different calculation scenarios for *I*_*blood*_(*x*) and *ØI*_*blood*_ by considering different proportions of the aorta and portal vein to the blood supply.

A portal vein–normalized iodine concentration *I*_*pv-n*_ was defined by only taking into consideration the iodine concentration of the portal vein for the blood-normalized iodine concentration of the liver parenchyma.

Accordingly, an aorta-normalized iodine concentration *I*_*a-n*_ was defined including only the iodine concentration of the aorta in the calculation of the blood-normalized iodine concentration of the liver parenchyma.

Finally, a mixed normalized iodine concentration *I*_*mixed-n*_ was defined by considering the blood supply of both vessels. Since the hepatic portal vein delivers approximately 75% of the blood flow to the liver and the hepatic artery supplies the resting 25% [33], we calculated the absolute iodine concentration of the mixed hepatic blood supply as:

3 $${I}_{mixed\_blood}\left(x\right)=0.75* {I}_{portalvein}\left(x\right)+0.25*{I}_{aorta}\left(x\right)$$

The three different blood-normalized iodine concentrations were obtained according to Eq. (2) as follows.

4 $${I}_{pv-n}\left(x\right)=\frac{{I}_{abs}\left(x\right)}{{I}_{portalvein}\left(x\right)}*{\varnothing I}_{portalvein}$$

5 $${I}_{a-n}\left(x\right)=\frac{{I}_{abs}\left(x\right)}{{I}_{aorta}(x)}*{\varnothing I}_{aorta}$$

$${I}_{mixed-n}(x)=\frac{{I}_{abs}\left(x\right)}{{I}_{mixed\_blood}\left(x\right)}*{\varnothing I}_{mixed\_blood}$$

6 $$=\frac{{I}_{abs}\left(x\right)}{0.75* {I}_{portalvein}\left(x\right)+0.25*{I}_{aorta}\left(x\right)}*\left(0.75*\varnothing {I}_{portalvein}+0.25*\varnothing {I}_{aorta}\right)$$

### Calculation of Thresholds for Absolute and Blood-normalized Iodine Uptake for Diagnosis of Hepatic Steatosis

We calculated the sensitivity, specificity, accuracy, positive predictive value (PPV), and negative predictive value (NPV) and analyzed different threshold levels for diagnosing hepatic steatosis by the quantitative iodine concentration. A total of 9 threshold levels were analyzed, with threshold values corresponding to 10, 20, 30, 40, 50, 60, 70, 80, and 90% of the gap between the average iodine concentration of healthy and fatty liver.

The thresholds were calculated as:

7 $$Threshold\;\left(x \%\right)= {\varnothing I}_{hepatic steatosis}+\frac{x*({\varnothing I}_{healthy\;liver}-{\varnothing I}_{hepatic\;steatosis})}{100}$$

### Statistical Analysis

Statistical analysis was performed using IBM SPSS Statistics, v.24.0. Differences between iodine concentration in healthy and fatty liver ROIs were assessed by independent samples *t*-test. Diagnostic performance was assessed by receiver operating characteristic (ROC) curve analysis. All tests were two-tailed, and a *p*-value < 0.05 was considered statistically significant.

## Results

### Demographics of the Study Population

The initial study population included a total of 292 patients. Seven patients were excluded due to insufficient image quality and artifacts (*n* = 7), 34 were excluded due to alternative diagnosis (hemihepatectomy (*n* = 2), splenectomy (*n* = 4), liver metastasis in segments 3, 4b, or 7 (*n* = 4), cirrhosis (*n* = 21), intrahepatic cholestasis (*n* = 2), and intrahepatic air (*n* = 1)), resulting in a final study population of 251 patients (mean age, 60 years ± 16; 134 male), 79 of which were diagnosed with hepatic steatosis by the reference standard method (Fig. 1 and Table 1).

**Table 1:** Characteristics of the final study population and of the different patient groups

Characteristics of study population						
		Amount	Min. Age	Max. Age	*ø* Age	*σ* Age
Healthy liver	Male patients	89	19	91	57.78	16.49
	Female patients	83	16	86	57.39	16.49
	All healthy patients	172	16	91	57.59	16.49
Hepatic steatosis	Male patients	45	26	92	63.02	13.91
	Female patients	34	28	86	61.77	13.14
	All patients with HS	79	26	92	62.48	13.60
Total study population		251	16	95	59.63	15.84

*HP* Hepatic Steatosis

### Relation Between Hepatic Steatosis and Absolute Iodine Concentration

The average attenuation difference between liver and spleen parenchyma was 42.3 HU (*σ* = 14.7 HU) for all patients diagnosed with hepatic steatosis and 7.0 HU (*σ* = 7.2 HU) for all patients with healthy liver parenchyma in our study population.

The absolute iodine concentration of the liver tissue was significantly higher for healthy liver tissue than for liver tissue identified to be affected by hepatic steatosis (*p* < 0.001). The mean value of absolute iodine concentration of all patients with healthy liver was 2.103 mg/ml (*σ* = 0.507 mg/ml). The mean value of absolute iodine concentration of all patients with fatty liver disease was 1.383 mg/ml (*σ* = 0.557 mg/ml) (Table 2).

**Table 2:** Absolute and blood-normalized iodine concentration values of healthy and fatty liver tissue

Iodine concentration values				
	Healthy liver		Hepatic steatosis	
	*ø*	*σ*	*ø*	*σ*
*I*_*abs*_ [mg/ml]	2.103	0.507	1.383	0.557
*I*_*pv-n*_ [mg/ml]	2.030	0.320	1.292	0.364
*I*_*a-n*_ [mg/ml]	2.006	0.383	1.356	0.384
*I*_*mixed-n*_ [mg/ml]	2.017	0.309	1.300	0.354

*I*_*abs*_ absolute iodine concentration, *I*_*pv-n*_ portal vein normalized iodine concentration, *I*_*a-n*_ aorta normalized iodine concentration, *I*_*mixed-n*_ mixed blood normalized iodine concentration

**Fig. 3:**
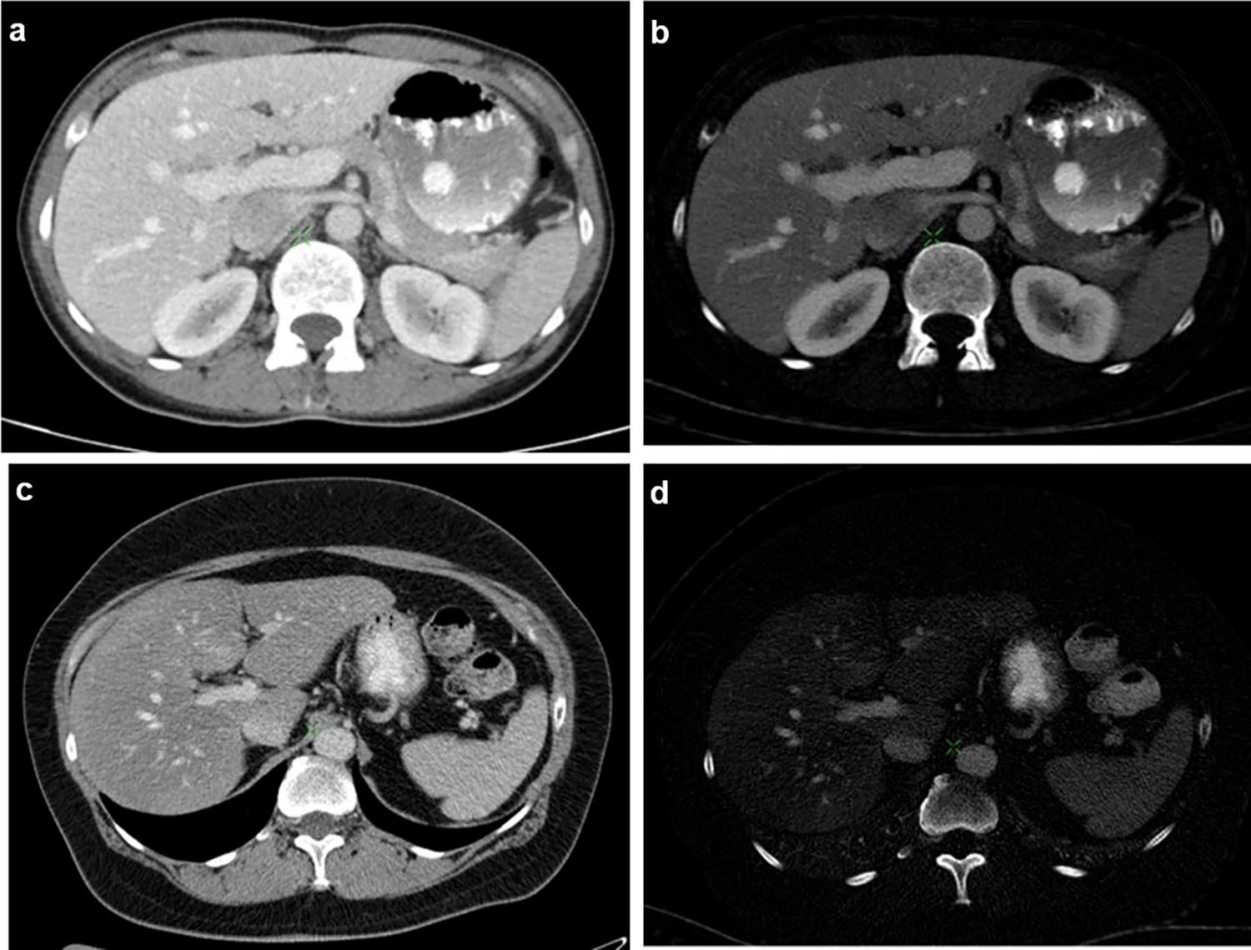
Portal venous phase DECT images of the upper abdomen in transverse plane. **a**, **c** One hundred twenty-kilovoltage peak HU images. **b**, **d** Material density iodine concentration images. The images in the upper row present the liver parenchyma of a 52-year-old male, healthy patient (CT value: 111 HU; iodine value: 2.75 mg/ml). In the lower row, DECT of the liver of a 51-year-old male patient with hepatic steatosis is shown, with lower CT value (68 HU) and decreased iodine value (0.88 mg/ml)

Figure 3 shows an example of the difference between the iodine uptake of healthy and fatty liver parenchyma in quantitative iodine images.

**Fig. 4:**
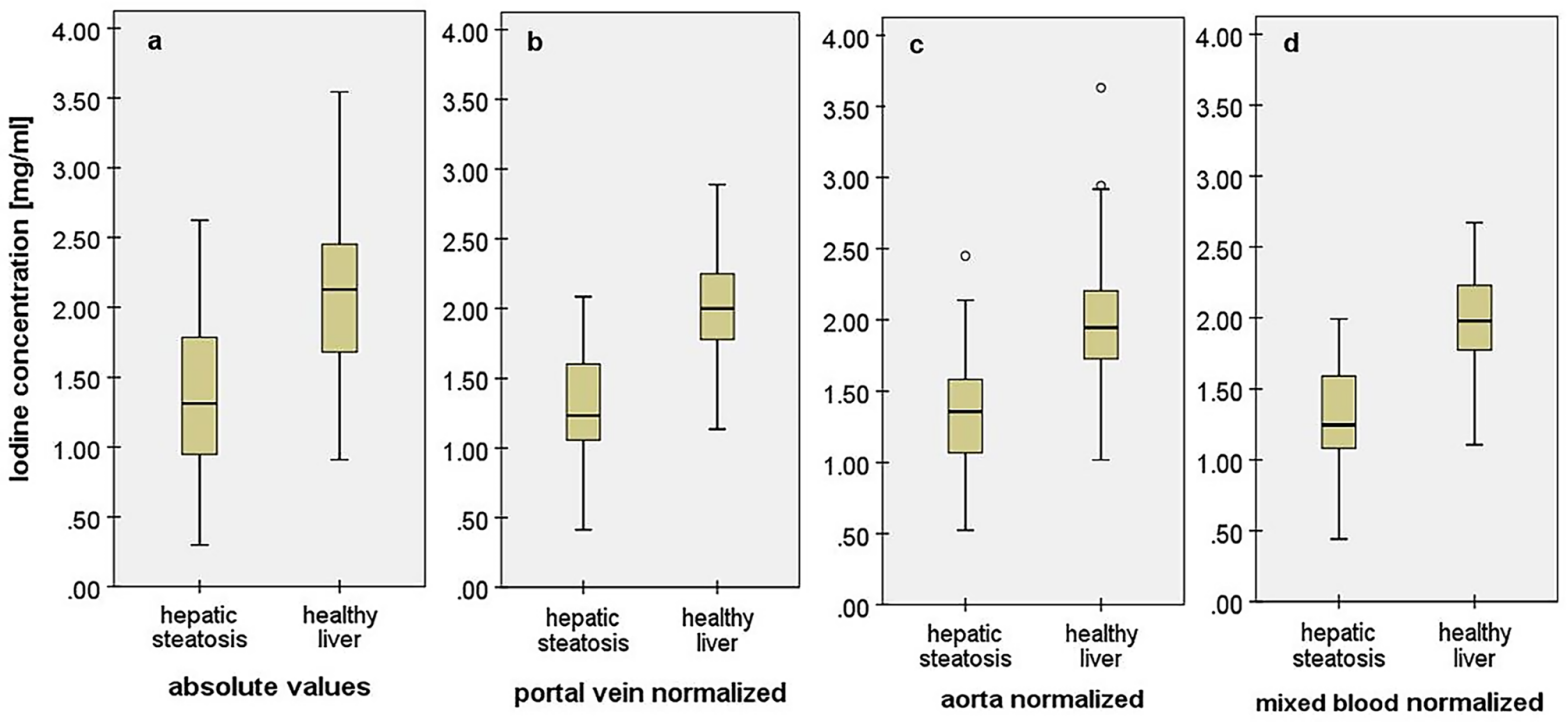
Different methods of iodine concentration value analysis of the liver **a** quantitative absolute iodine concentrations *I*_*abs*_, **b** portal vein normalized iodine concentration *I*_*pv-n*_, **c** aorta normalized iodine concentration *I*_*a-n*_, and **d** mixed blood-normalized iodine concentration *I*_*mixed-n*_. All four analyzed iodine concentrations show significant differences between healthy liver and hepatic steatosis (*p* < 0.001)

Figure 4a shows the box plot diagrams comparing the absolute iodine concentrations of healthy and fatty liver.

### Relation Between Hepatic Steatosis and Blood-normalized Iodine Concentrations

Significant differences could also be found for all three blood-normalized iodine concentrations between healthy and fatty liver tissue (*p* < 0.001 for all three blood-normalized values).

The mean values of all patients with healthy liver tissue and the corresponding standard deviations were 2.030 mg/ml (*σ* = 0.320 mg/ml), 2.006 mg/ml (*σ* = 0.383) mg/ml, and 2.017 mg/ml (*σ* = 0.309 mg/ml) for *I*_*pv-n*_, *I*_*a-n*_, and *I*_*mixed-n*_, respectively.

The mean values of all patients with hepatic steatosis and the corresponding standard deviations were 1.292 mg/ml (*σ* = 0.364 mg/ml), 1.356 mg/ml (*σ* = 0.384 mg/ml), and 1.300 mg/ml (*σ* = 0.354 mg/ml) for *I*_*pv-n*_, *I*_*a-n*_, and *I*_*mixed-n*_, respectively.

The overall mean values and standard deviations for the three blood-normalized iodine concentrations for patients with and without hepatic steatosis are shown in Table 2.

Figure 4b–c show the box plot diagrams comparing the three different blood-normalized iodine concentrations of healthy and fatty liver of the total study population.

### Definition of Appropriate Thresholds for Absolute and Blood-normalized Iodine Uptake for Diagnosis of Hepatic Steatosis

For each of the four measurement methods, we analyzed nine different threshold levels, equally distributed between the average iodine concentration value for healthy and fatty liver respectively, and calculated the quantitative threshold iodine concentrations correspondingly. Table 3 shows the calculated thresholds for absolute iodine concentration and blood-normalized iodine concentrations. The results for sensitivity, specificity, accuracy, PPV, and NPV, corresponding to each of the defines thresholds, are shown in Table 4.

**Table 3:** Average iodine concentrations of healthy and fatty liver and calculated potential thresholds for diagnosis of hepatic steatosis

Threshold levels for diagnosis of hepatic steatosis											
	Average values		Thresholds								
	Hepatic steatosis	Healthy liver	10%	20%	30%	40%	50%	60%	70%	80%	90%
*I*_*abs*_	1.383	2.103	1.455	1.527	1.599	1.671	1.743	1.815	1.887	1.959	2.031
*I*_*pv-n*_	1.292	2.030	1.366	1.440	1.514	1.587	1.661	1.735	1.808	1.882	1.956
*I*_*a-n*_	1.356	2.006	1.421	1.486	1.551	1.616	1.681	1.746	1.811	1.876	1.941
*I*_*mixed-n*_	1.300	2.017	1.372	1.443	1.515	1.587	1.658	1.730	1.802	1.874	1.945

*I*_*abs*_ absolute iodine concentration, *I*_*pv-n*_ portal vein normalized iodine concentration, *I*_*a-n*_ aorta normalized iodine concentration, *I*_*mixed-n*_ mixed blood normalized iodine concentration

**Table 4:** Diagnostic performance for each threshold level

Diagnostic performance of threshold levels										
		Thresholds								
		10%	20%	30%	40%	50%	60%	70%	80%	90%
*I*_*abs*_	Sensitivity (%)	62	65	67	72	73	78	80	84	**87**
	Specificity (%)	**89**	87	82	76	74	69	66	58	54
	Accuracy (%)	**80**	**80**	77	75	74	72	70	66	65
	PPV (%)	**72**	70	63	58	56	53	52	48	47
	NPV (%)	84	84	84	86	86	87	88	88	**90**
*I*_*pv-n*_	Sensitivity (%)	67	70	72	77	89	90	92	94	**97**
	Specificity (%)	**99**	96	94	90	85	79	72	67	56
	Accuracy (%)	**89**	88	87	86	86	82	78	75	69
	PPV (%)	**96**	89	85	77	74	66	60	56	51
	NPV (%)	87	87	88	90	94	94	95	96	**98**
*I*_*a-n*_	Sensitivity (%)	51	62	66	76	84	87	91	92	**94**
	Specificity (%)	**99**	97	92	85	80	73	66	59	48
	Accuracy (%)	84	**86**	84	82	81	78	74	69	63
	PPV (%)	**95**	91	79	71	66	60	55	51	45
	NPV (%)	81	85	85	89	91	93	94	94	**94**
*I*_*mixed-n*_	Sensitivity (%)	63	68	70	73	85	90	92	94	**99**
	Specificity (%)	**99**	**99**	97	92	86	80	72	63	56
	Accuracy (%)	88	**90**	88	86	86	83	78	73	69
	PPV (%)	**98**	**98**	92	81	74	68	60	54	51
	NPV (%)	86	87	87	88	93	95	95	96	**99**

The bold emphasis indicates the best results in terms of sensitivity, specificity, accuracy, PPV and NPV obtained with each of the evaluated iodine concentrations

*I*_*abs*_ absolute iodine concentration, *I*_*pv-n*_ portal vein normalized iodine concentration, *I*_*a-n*_ aorta normalized iodine concentration, *I*_*mixed-n*_ mixed blood normalized iodine concentration, *PPV* positive predictive value, *NPV* negative predictive value

**Fig. 5:**
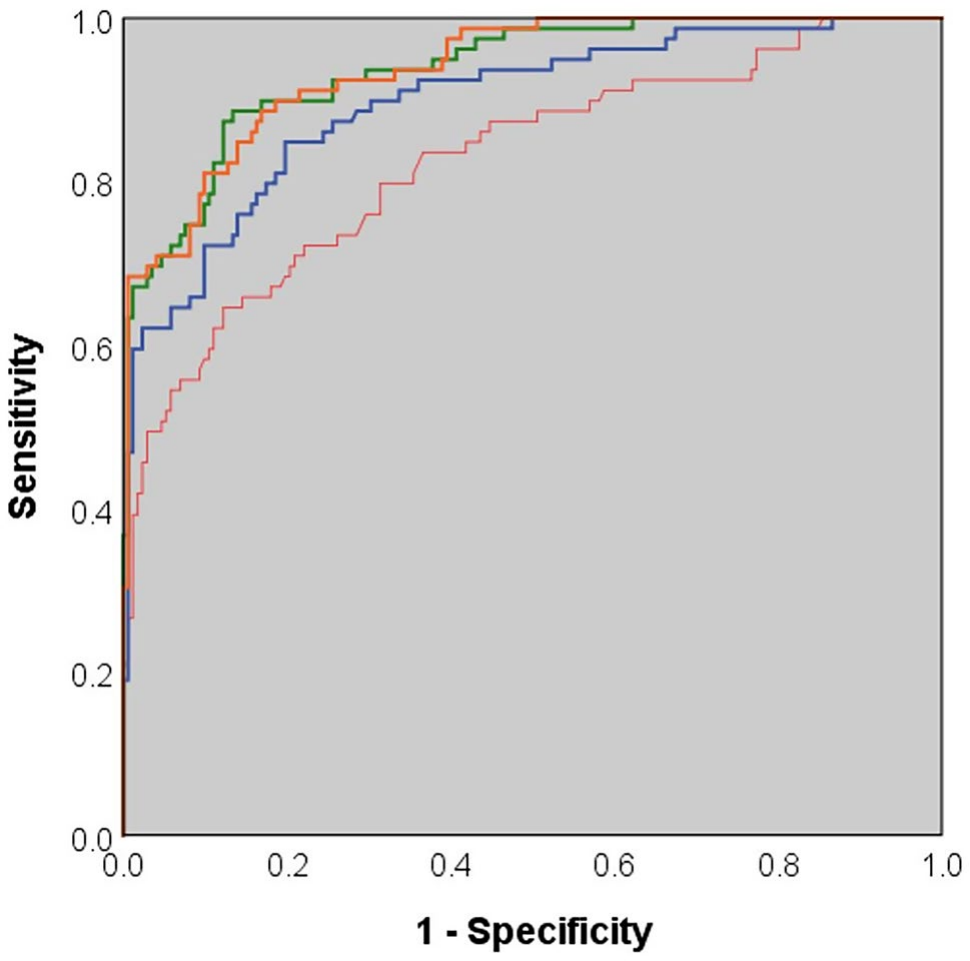
Comparison of ROC curves for (red) absolute iodine concentration *I*_*abs*_, (green) portal vein normalized iodine concentration *I*_*pv-n*_, (blue) aorta normalized iodine concentration *I*_*a-n*_, and (orange) mixed blood-normalized iodine concentration *I*_*mixed-n*_. Highest diagnostic performance was obtained by *I*_*pv-n*_ and *I*_*mixed-n*_

Figure 5 shows the ROC curve for the different measurement methods. The best results were obtained for the portal vein and the mixed blood-normalized iodine concentration (AUC = 0.937 ± 0.016, 95%-KI: 0.906–0.968 and 0.937 ± 0.015, 95%-KI: 0.908–0.967, respectively). The AUC was 0.896 ± 0.022 (95%-KI: 0.853–0.940) for aorta-normalized iodine concentration and 0.825 ± 0.030 (95%-KI: 0.767–0.884) for absolute iodine concentration.

Considering the overall results for sensitivity, specificity, PPV, and NPV, for blood-normalized iodine values, best results were obtained with a threshold at 50% of the gap between healthy and fatty liver iodine concentrations, considering well-balanced values for sensitivity and specificity. In absolute numbers, this yields a threshold of 1.661 mg/ml for *I*_*pv-n*_ (sensitivity: 89%, specificity: 85%, accuracy: 86%, PPV: 74%, NPV: 94%), 1.681 mg/ml for *I*_*a-n*_ (sensitivity: 84%, specificity: 80%, accuracy: 81%, PPV: 66%, NPV: 91%), and 1.658 mg/ml for *I*_*mixed-n*_ (sensitivity: 85%, specificity: 86%, accuracy: 86%, PPV: 74%, NPV: 93%). For absolute iodine concentration, best results were obtained at a threshold at 40% in terms of overall results and balanced sensitivity and positivity (threshold: 1.671 mg/ml, sensitivity: 72%, specificity: 76%, accuracy: 75%, PPV: 58%, NPV: 86%).

## Discussion

The diagnosis of hepatic steatosis with CT has traditionally been performed with unenhanced images. The diagnosis with contrast-enhanced CT images has been often considered to be less precise [11–15], since the magnitude of contrast enhancement is commonly measured by analyzing the HU values of a tissue, allowing only an approximate information about the contrast medium uptake, since it includes the superposition of contrast medium and body tissue and depends on the injection protocol and patient physiology. However, in the general clinical practice, many CT examinations are performed using contrast administration, since the information provided by contrast enhancement is essential for the discrimination of different liver lesions. Therefore, the diagnosis of hepatic steatosis in contrast-enhanced CT images should not be neglected, and evaluation of absolute iodine values may improve the diagnosis of steatosis in contrast enhanced CT.

In this study, we present an analysis of average values for quantitative iodine concentrations of liver tissue affected by hepatic steatosis in contrast-enhanced portal venous phase quantitative iodine images in DECT.

The results of the measurements showed significant differences concerning the iodine uptake between healthy liver and fatty liver parenchyma. This applies to both the absolute iodine concentration values and the blood-normalized iodine concentration values. The measured iodine concentrations were significantly lower in the liver parenchyma of patients with hepatic steatosis than of healthy liver patients (*I*_*abs*_: 1.383 mg/ml ± 0.557 vs. 2.103 mg/ml ± 0.507, *p* < 0.001). We hence deduce that fatty liver tissue has a reduced capacity of iodine uptake in comparison to healthy liver tissue.

Blood normalization reduced the influence of variations in the contrast agent administration protocol and of patient-specific physiological and anatomical singularities (i.e., hemodynamics, blood volume, cardiac output, etc.), and therefore allowed a more precise evaluation of the actual iodine uptake of the liver parenchyma itself, which is reflected in the higher diagnostic performance of blood-normalized iodine values (AUC = 0.937 ± 0.016 for *I*_*pv-n*_ vs. AUC = 0.825 ± 0.030 for *I*_*abs*_) and also in a smaller standard deviation of the average iodine concentration values for healthy liver and fatty liver (*σ*(*I*_*abs*_): 0.507 mg/ml and 0.557 mg/ml vs. *σ*(*I*_*pv-n*_): 0.320 mg/ml and 0.364 mg/ml for healthy and fatty livers, respectively, *p* < 0.001).

We thus consider the results for blood-normalized iodine concentration values to have a higher diagnostic relevance than those for absolute iodine concentrations. The best results concerning sensitivity, specificity, accuracy, PPV, and NPV were obtained for the portal vein and mixed blood-normalized iodine concentrations.

Depending on the used threshold level, higher specificity or sensitivity levels may be reached for the diagnosis of hepatic steatosis at the cost of an impaired balance.

The significant differences and the diagnostic performance observed in the analysis of blood-normalized quantitative iodine concentrations therefore provide a new possible biomarker for the diagnosis of hepatic steatosis in contrast-enhanced DECT and might even provide advantages concerning the diagnostic accuracy of hepatic steatosis compared to the current gold standard of relative HU values.

There are several limitations to this study. First, the reference standard for diagnosis of hepatic steatosis and healthy liver tissue was based on the original contrast-enhanced images that were the source for the computerized generation of the iodine images subject to analysis. Further prospective studies should be performed with a clinical diagnosis or ideally a histologic finding as a reference standard to corroborate the results of our work.

The defined average values and thresholds, particularly those referring to absolute iodine concentrations, are conditioned by the CT protocol used, since the type and amount of contrast agent, the method of contrast administration, and the imaging delay have an essential impact on the measured iodine concentration. This may consequently affect the comparability of the presented values with values obtained by other studies or centers.

Furthermore, the dual-energy technology used may influence the estimation of iodine concentration values and systemic variations may occur during the quantitative iodine measurements by DECT affecting the accuracy of the resulting values. Also, the measurements could be influenced by scanning protocols or patient size. Nevertheless, these issues have been analyzed in the previous publications, which have concluded that they are expected to cause only a minor impact on the results [23–26, 32].

Finally, PPV and NPV depend on the prevalence of a disease in a study population. Therefore, the values presented in this study may vary for other study cohorts.

## Conclusion

Blood-normalized iodine concentrations can provide a potential biomarker for diagnosis of hepatic steatosis in contrast-enhanced dual-energy CT. A threshold of 1.661 mg/ml for portal vein–normalized iodine concentrations can help to quantitatively diagnose hepatic steatosis. Since this study presents a first analysis of quantitative iodine concentration values of the liver tissue affected by hepatic steatosis, future prospective studies with further patient cohorts and correlated histological findings should be performed to validate the presented results.
